# The Effect of Repetitive Transcranial Magnetic Stimulation Treatment on Plasma BDNF Concentration and Executive Functions in Parkinson’s Disease: A Theoretical Translational Medicine Approach

**DOI:** 10.3390/ijms26031205

**Published:** 2025-01-30

**Authors:** Gianna Carla Riccitelli, Riccardo Gironi, Giorgia Melli, Alain Kaelin-Lang

**Affiliations:** 1 Non-Invasive Brain Stimulation Research Unit, Institute of Clinical Neurocenter of Southern Switzerland, EOC, 6900 Lugano, Switzerland; riccardo.gironi@eoc.ch (R.G.);; 2 Faculty of Biomedical Sciences, Università della Svizzera Italiana, 6900 Lugano, Switzerland; giorgia.melli@eoc.ch; 3 Neurodegenerative Diseases Group, Laboratory for Translational Research EOC, 6500 Bellinzona, Switzerland; 4 Movement Disorders Unit, Institute of Clinical Neurocenter of Southern Switzerland, EOC, 6900 Lugano, Switzerland; 5 Department of Neurology, Inselspital, Bern University Hospital, 3010 Bern, Switzerland

**Keywords:** neuroprotection, biomarker validation, translational medicine, non-invasive therapy

## Abstract

Parkinson’s disease (PD) neuropathology is marked by the selective loss of dopaminergic neurons in the substantia nigra pars compacta, accompanied by the widespread involvement of central and peripheral structures. Brain-derived neurotrophic factor (BDNF), a neurotrophin crucial for the survival of dopaminergic neurons, plays a pivotal role in neuronal and glial development, neuroprotection, and the modulation of synaptic plasticity. Repetitive transcranial magnetic stimulation (rTMS), a non-invasive technique, enhances neurotransmitter release, trans-synaptic efficacy, signaling pathways, gene transcription, neuroplasticity, and neurotrophism. Evidence supports that high-frequency rTMS increases BDNF expression and improves task-specific cognitive deficits in PD patients. This article outlines a detailed protocol to investigate whether rTMS targeting the dorsolateral prefrontal cortex bilaterally induces changes in plasma BDNF levels, the plasma-derived exosomal BDNF concentration, and executive functions in individuals with PD. Identifying non-invasive interventions that effectively modulate the neurobiological mechanisms underlying cognitive and behavioral functions is critical for addressing cognitive impairments and mitigating disease progression in the PD population. This study aims to advance translational research by identifying biomarkers and developing therapeutic strategies for future applications in neurodegenerative diseases.

## 1. Introduction

Parkinson’s disease (PD) is one of the most common neurodegenerative diseases, second only to Alzheimer’s disease (AD) [1], characterized by motor and non-motor symptoms including cognitive deficits such as executive dysfunction, which worsen with disease progression and contribute significantly to patient burden.

These deficits are linked to the degeneration of dopaminergic (DAergic) neurons in the nigrostriatal pathway, which is more pronounced in PD patients with mild cognitive impairment (PD-MCI) than in those without cognitive abnormalities. However, PD-MCI patients had the relative preservation of other dopaminergic systems in the brain compared to PD patients with dementia [2], making PD-MCI patients more suitable for focused rehabilitative approaches.

Neurotrophins are endogenous proteins responsible for the development, function, and survival of neurons [3]. The expression of neurotrophins in the brain is downregulated in postmortem PD patients [4,5], and the supplementation of neurotrophin has demonstrated neuroprotective effects in animal studies on PD and clinical trials [6].

Among these neurotrophins, brain-derived neurotrophic factor (BDNF) has garnered significant attention due to its role in dopaminergic neuron survival, synaptogenesis, and plasticity [7]. It supports the survival and function of DAergic neurons by increasing the number of tyrosine hydroxylase (TH)-positive neurons and enhancing dopamine secretion [8] in the striatum and substantia nigra [9,10]. 

Notably, reduced BDNF expression in the substantia nigra and striatum in PD patients has been implicated in disease progression, with the presence of low blood BDNF levels emerging as a potential biomarker for disease severity [4,5].

BDNF plays a key role in cognition, with reduced activity-dependent expression impairing executive functions (EFs) linked to hippocampal-prefrontal circuitry [11]. Impairment in this circuitry due to reduced BDNF expression can exacerbate cognitive deficits in PD. Studies, such as Angelucci et al. (2015), have demonstrated that cognitive rehabilitation can improve EFs and increase serum BDNF levels in PD-MCI patients, supporting the hypothesis that targeting BDNF could enhance cognitive outcomes in this population [12].

Emerging research has highlighted extracellular vesicles (EVs)—which mediate cell-to-cell communication and can cross the blood–brain barrier—as potential carriers of BDNF. These EVs, particularly exosomes, are critical for biomarker validation as they provide insight into central nervous system processes [13]. PD patients exhibit elevated plasma EV concentration and altered EV surface antigens, which correlate with disease severity and cognitive impairment [14].

Plasma exosomes, the smallest EVs, carry BDNF, making them ideal candidates for investigating neurotrophic changes associated with interventions like repetitive transcranial magnetic stimulation (rTMS) [15]. 

Specifically, low plasma exosomal BDNF levels have been associated with greater motor severity and functional impairments in PD patients, as reported by Chung et al. (2020). These findings underscore the importance of studying how rTMS-induced changes in BDNF levels, particularly those mediated by exosomes, might relate to clinical outcomes in PD [15]. Nevertheless, the role of EVs on the BDNF expression in PD warrants further investigation.

Repetitive transcranial magnetic stimulation (rTMS) is a non-invasive intervention that has been shown to regulate the expression of neurotrophic factors, including BDNF, by promoting neuronal differentiation, survival, and plasticity [16]. 

For instance, Choung et al. 2021 demonstrated that high-frequency (HF) rTMS enhanced BDNF expression in a model of Alzheimer’s disease alongside improvements in neural survival markers [15]. The neuroprotective and neuroplastic benefit of rTMS has been observed in studies across various neurological diseases including major depression [17], amyotrophic lateral sclerosis [18], and PD [19,20].

HF-rTMS targeting the dorsolateral prefrontal cortex (DLPFC)—a region crucial for EFs—has consistently shown promise in modulating BDNF expression and improving cognitive functions. EF abnormalities in PD are closely linked to frontal lobe damage, particularly in the DLPFC, which undergoes the progressive degradation of DAergic neurons [21].

Taken together, this evidence suggests that TMS has the potential to modulate plasma BDNF levels and exosomal BDNF concentrations, offering a non-invasive strategy to address both motor and cognitive symptoms in PD.

Our proposed protocol aims at investigate whether HF-rTMS treatment for 4 weeks, targeting the right and left DLPFC, can increase plasma BDNF and plasmatic EV-derived BDNF concentration and improve EFs in PD patients. 

## 2. Primary and Secondary Outcomes

### 2.1. Primary Outcomes

#### 2.1.1. Change in Plasma BDNF Levels

We will evaluate the changes in plasma BDNF levels after 4 weeks of rTMS treatment in patients with PD-MCI compared to PD-MCI patients receiving sham rTMS treatment and those receiving no rTMS treatment.

#### 2.1.2. Change in Plasmatic EV-Derived BDNF Concentration

We will assess the changes in plasmatic EV-derived BDNF concentrations after 4 weeks of rTMS treatment in PD-MCI patients relative to PD-MCI patients receiving sham rTMS treatment and those receiving no rTMS treatment.

#### 2.1.3. Change in EF Performance

We will measure the changes in executive function performance after 4 weeks of rTMS treatment in PD-MCI patients compared to PD-MCI patients receiving sham rTMS treatment and those receiving no rTMS treatment.

### 2.2. Secondary Outcomes

#### 2.2.1. Sustained Changes in Plasma BDNF Levels

We will assess the changes in plasma BDNF levels 8 weeks post rTMS treatment in PD-MCI patients compared to PD-MCI patients who have received sham rTMS treatment and those who have received no rTMS treatment.

#### 2.2.2. Sustained Changes in Plasmatic EV-Derived BDNF Concentration

We will evaluate the changes in plasmatic EV-derived BDNF concentrations 8 weeks post rTMS treatment in PD-MCI patients relative to PD-MCI patients receiving sham rTMS treatment and those receiving no rTMS treatment.

#### 2.2.3. Sustained Changes in EF Performance

We will determine the changes in EF performance 8 weeks post rTMS treatment in PD-MCI patients compared to PD-MCI patients receiving sham rTMS treatment and those receiving no rTMS treatment.

#### 2.2.4. Correlation Between BDNF Biomarkers and EF Performance

We will investigate the correlation between plasma BDNF levels or plasmatic EV-derived BDNF concentrations and EF scores after 4 weeks of rTMS treatment and 8 weeks post treatment in PD-MCI patients as compared to PD-MCI patients receiving sham rTMS treatment and those receiving no rTMS treatment.

## 3. Materials

### 3.1. Laboratory Measurements, Neurocognitive Assessment, and TMS Intervention

#### 3.1.1. Blood Sampling

All the blood samples will be drawn in the morning after overnight fasting at three time points: before the start of rTMS intervention (baseline), within 1 week after the end of treatment (T1), and within 1 week after the maintenance period (T2).

Blood will be drawn by trained staff from the antecubital vein (gauge of needle: 21 G × ¾″ × 7″, BD Vacutainer, REF 367282; BD, NJ, USA) into 15 vacutainers to ensure sufficient sample collection. Possible confounders for BDNF levels such as sex, age, and BMI will be accounted by statistical analysis and by analyzing multiple measurements at different time points intra subjects.

#### 3.1.2. Plasma BDNF Quantification

**Figure 1:**
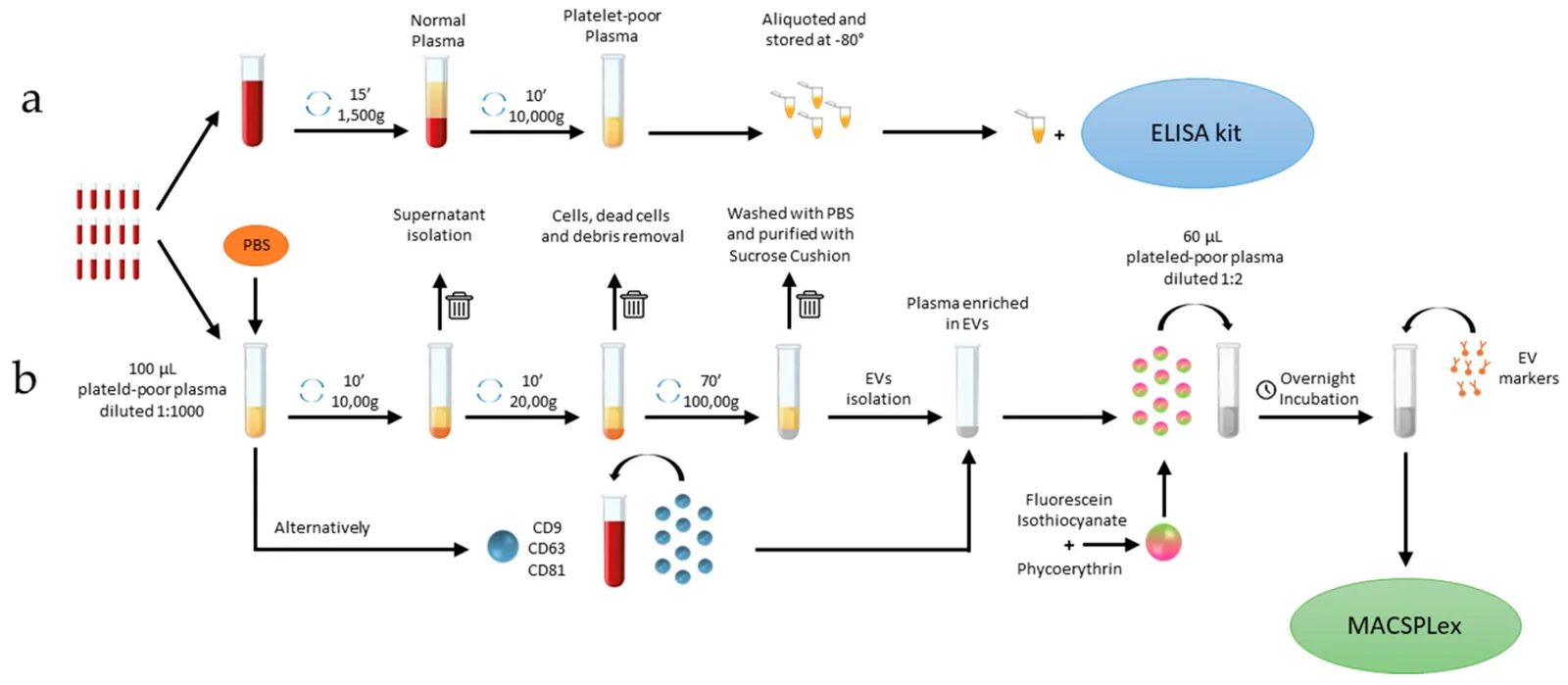
Laboratory workflow (**a**): plasma Brain-Derived Neurotrophic Factor (BDNF) quantification involves centrifugation to obtain platelet-poor plasma, followed by analysis using the mature BDNF Rapid ELISA kit; (**b**): plasma-derived extracellular vesicle (EV) isolation includes sequential centrifugation, ultracentrifugation, or immunomagnetic bead isolation to purify EVs. EV-enriched plasma will be stored at −80 °C for subsequent analysis using the MACSPlex Human Exosome Kit to detect EV-specific markers.

1. Centrifugation
  - Blood samples will be centrifuged at 4 °C for 15 min at 1500× *g* to isolate normal plasma;
  - To obtain platelet-poor plasma, five plasma samples will undergo a second centrifugation step at 10,000× *g* for 10 min at 4 °C [22].
2. Storage
  - After centrifugation, plasma samples will be aliquoted into Eppendorf tubes (Eppendorf SE, Hamburg, Germany) in single-use volumes and stored at −80 °C until analysis.
3. Analysis
  - Plasma samples will be analyzed using the mature BDNF Rapid ELISA kit (Cat. # BEK-221, Biosensis, Thebarton, Australia) following the manufacturer’s instructions (Figure 1a);
  - A dilution factor of 1:20 will be used, based on previous findings by Gejl et al. (2019), for optimal detection [22] (Figure 1a).

#### 3.1.3. Plasma-Derived EV Isolation

Plasma-derived extracellular vesicles (EVs) will be isolated following the state-of-the-art procedures according to MISEV 2023 guidelines [23], with key steps detailed below [24] (Figure 1b):

1. Sample preparation
  - Ten milliliters of platelet-poor plasma will be diluted (1:1000) with PBS to reduce viscosity and facilitate isolation.
2. Sequential Centrifugation
  - Centrifugation will be performed at 10,000× *g* for 10 min at 4 °C;
  - The supernatant will be transferred to a new container and centrifuged at 20,000× *g* for 10 min at 4 °C to remove cells, dead cells, and debris;
  - EVs will be pelleted by ultracentrifugation at 100,000–110,000× *g* for 70 min at 4 °C. The pellet will be washed with PBS to remove contaminants and resuspended in a small volume of PBS.
3. Sucrose Cushion
  - For additional purification, exosomes will be floated on a 30% sucrose cushion to eliminate nonspecific protein aggregates and other contaminants.
4. Immunomagnetic Beads
  - Alternatively, magnetic beads coated with antibodies targeting exosome-specific surface markers (e.g., CD9, CD63, CD81) will be used to isolate EVs directly without ultracentrifugation.
5. Storage
  - EV-enriched plasma will be aliquoted into single-use tubes and stored at −80 °C until further analysis.

#### 3.1.4. EV Characterization

1. Detection of EV-Specific Markers
  - The MACSPlex Human Exosome Kit (Miltenyi, Bergisch Gladbach, Germany) will be used. This kit employs 4.8 μm polystyrene beads conjugated with capture antibodies for EV markers;
  - Platelet-poor plasma (60 µL) will be diluted 1:2 in buffer solution and incubated with beads overnight.
2. Analysis
  - EVs bound to beads will be detected using APC-conjugated antibodies and analyzed on a MACSQuant Analyzer-10 flow cytometer (Miltenyi, Bergisch Gladbach, Germany);
  - A blank control (MACSPlex Buffer + beads + detection antibodies) will measure background signal;
  - Median fluorescence intensity (MFI) for BDNF will be normalized against EV markers (CD9, CD63, CD81) to calculate the normalized MFI (nMFI).
3. Reliability Testing
  - To ensure specificity, EV enrichment using ultracentrifugation will be compared with samples processed without enrichment.

#### 3.1.5. Quality Control

1. Additional Characterization
  - Nanoparticle Tracking Analysis (NTA) or Dynamic Light Scattering (DLS) will measure nanoparticle size and concentration;
  - Western blotting for EV markers (TSG101, Alix, Synthenin) will complement the MACSPlex analysis.
2. Statistical Validation
  - nMFI values will be statistically analyzed across experimental groups, ensuring reproducibility and reliability.

### 3.2. Neurocognitive Assessment

To exclude an operator-dependent effect, the same neuropsychologist will conduct the assessment within subjects and will be blind to group membership to maintain the validity and integrity of the assessment.

Cognitive profile will be investigated with the Behavioural Assessment of the Dysexecutive Syndrome (BADS). It is a comprehensive tool for assessing executive functioning through tasks that simulate real-life scenarios. BADS is particularly effective in identifying executive dysfunctions in patients with brain injuries or neurological conditions such as PD [25].

The impact of PD on patient’s daily life will measured with the self-reported Parkinson’s Disease Questionnaire (PDQ-39) [26].

### 3.3. TMS Intervention

In this project, a two-site rTMS stimulation delivered by a Magstim unit (Magstim Co., Whitland, UK) featuring a double 70 mm cooled coil will be applied.

R-Gr will receive 4 weeks of excitatory rTMS stimulation of the left and right DLPFC; S-Gr will receive 4 weeks of rTMS sham treatment of the left and right DLPFC; and C-Gr will be free of rTMS treatment. For R-Gr and S-Gr, each week of rTMS treatment will consist of five sessions (30 min each [15 min for each region], one per day).

For each target area, the stimulation protocol will comprise 1500 pulses at 15 Hz, formatted as a 3 s train, followed by a 28 s inter-interval at 100% motor threshold (MT) that will be delivered. 

A 15 Hz stimulation frequency will be used, based on its established efficacy in enhancing neuroplasticity and modulating BDNF levels [27,28] including in PD [29].

The stimulation protocol consists of 1500 pulses per hemisphere, applied in a 3-s train, followed by a 28-s inter-train interval. This structure balances efficacy while minimizing participant fatigue and the risk of adverse effects.

The intensity will be set at 100% of each participant’s resting MT to ensure consistent stimulation effects while accounting for individual variability [30]. Adjustments will be made if participants report discomfort. 

Individualized coil positioning will be guided using T1-weighted magnetic resonance imaging MRI volume (sequence parameters: TR = 1900 ms; TE = 2.1 ms; TI = 900 ms; FOV = 240 mm^2^; matrix = 256 × 256; voxel size = 0.9 × 0.9 × 0.9 mm^3^) to accurately localize the DLPFC bilaterally. 

For each site target, the coil will be placed with the junction of the two coil wings above the target point.

Sham stimulation will use the same parameters as real sessions, with a sham coil that mimicks operational noise without delivering actual stimulation.

In both real and sham treatment, the coil position over right and left DLPFC will be constantly monitored using the Softaxic neuronavigation system (Electro Medical Systems, Bologna, Italy) coupled with a Polaris Vicra infrared camera. Each session will last for about 45 min including time for set up and 30 min of stimulation.

### 3.4. Safety Consideration

The rTMS protocol adheres to International Federation of Clinical Neurophysiology safety guidelines.

Adverse Event Monitoring: Participants will be monitored for side effects, such as headaches or scalp discomfort, using standardized reporting tools. Any adverse events will be recorded and managed according to predefined criteria.

Stopping Criteria: If participants experience significant discomfort or side effects that compromise their well-being, predefined stopping criteria will be implemented to ensure safety.

## 4. Experimental Procedures

### 4.1. Project Design

This will be a prospective, double-blind, randomized, controlled single-center study. PD patients that satisfy inclusion and exclusion criteria will be randomly assigned to one of three groups: (1) real group (R-Gr), in which the patients will receive 4 weeks of real rTMS stimulation; (2) sham group (S-Gr), in which the patients will receive 4 weeks of sham treatment of rTMS stimulation; and (3) control group (C-Gr), in which the patients will be free of treatment. Here, participants will receive no rTMS treatment, and this group will serve as a baseline comparator. This group is designed to control for placebo effects and participants’ expectations, which are known to influence outcomes inducing striatal DA release in PD patients [31,32,33].

Each week of rTMS treatment will consist of five sessions (45 min each, one per day).

Blood collection and neurocognitive investigation will be performed at baseline (T0), after 4 weeks from baseline and 4 weeks of rTMS treatment (T1), and 8 weeks after the end of treatment (maintenance period [T2]).

### 4.2. Recruitment and Screening Procedure

**Figure 2:**
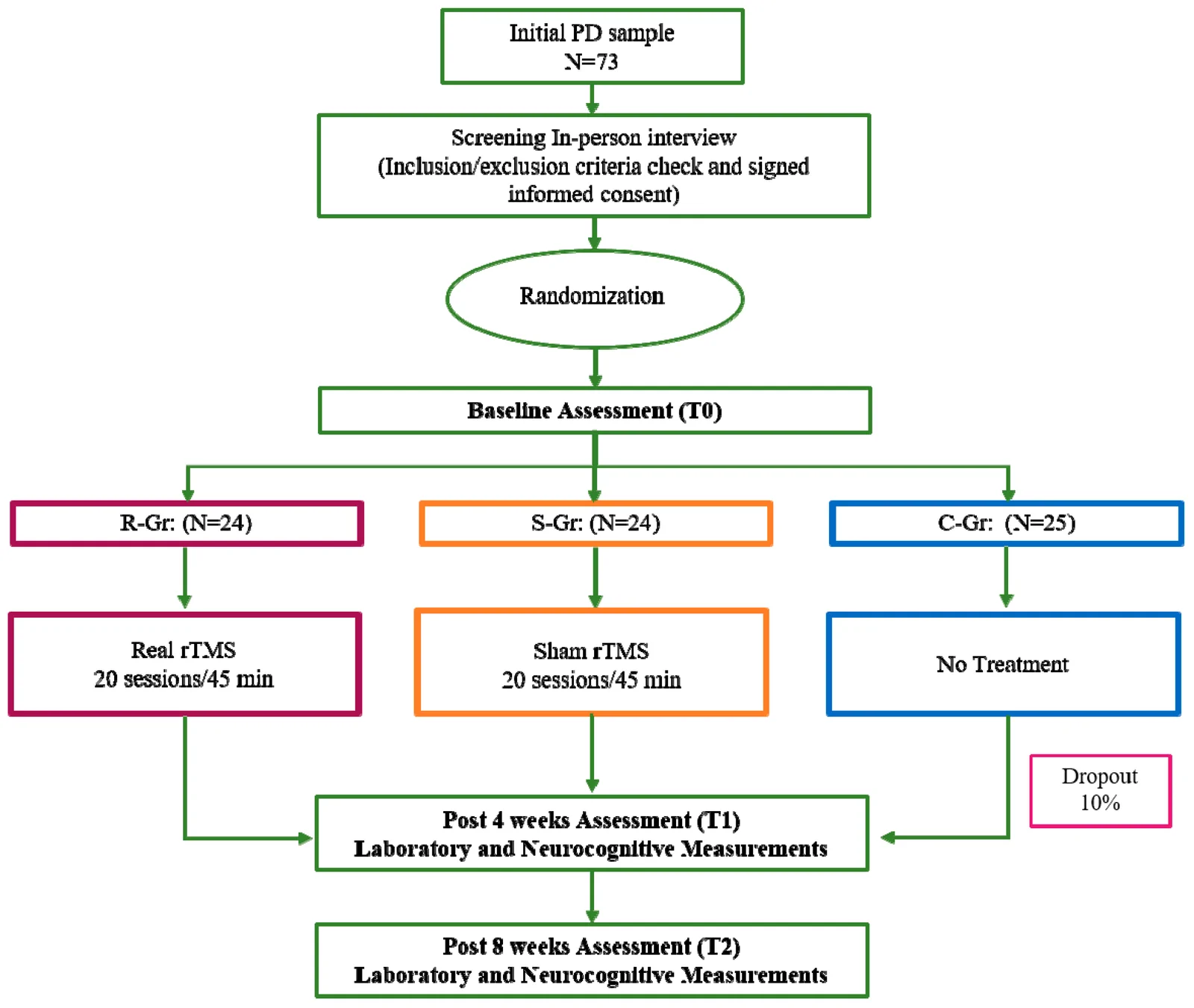
Study flowchart. The study will involve 73 participants diagnosed with PD, who are randomized into three groups: R-Gr (24 patients receiving real rTMS), S-Gr (24 patients receiving sham rTMS), and C-Gr (25 control participants without rTMS). Assessments, including blood collection, neurocognitive evaluation, and functional testing, will be conducted at baseline (T0), after 4 weeks of intervention (T1), and at 8-week follow-up (T2). Participants in the rTMS groups will undergo a total of 20 sessions, each lasting 45 min. A 10% dropout rate is anticipated. Abbreviations: PD = Parkinson’s disease; rTMS = repetitive transcranial magnetic stimulation; R-Gr = real group; S-Gr = sham group; C-Gr = control group.

Patients will be recruited from the movement disorder outpatient clinic at Neurocenter of Southerland Switzerland EOC, in Lugano. Graphical representation of the sequence of events is illustrated in Figure 2.

At screening, we will evaluate all aspects useful to satisfy the inclusion/exclusion criteria of the study. In this context, PD patients that satisfy Litvan’s diagnostic criteria for MCI [34] will undergo a clinical interview and questionnaires to ensure their ability to understand the instructions and to exclude the most severely depressed subjects. 

### 4.3. Main Inclusion and Exclusion Criteria

#### 4.3.1. Inclusion Criteria

Participants will be eligible for inclusion if they meet the following criteria:

1. Age range
  - Participants must be aged between 50 and 75 years, a range chosen to reflect the population most affected by PD-related cognitive decline while minimizing age-related confounding factors.
2. Disease severity
  - A definitive clinical diagnosis of PD in accordance with the UK Parkinson’s Disease Society Brain Bank criteria [35];
  - The severity of PD will be assessed using the Hoehn and Yahs scale, with eligible participants having a disease stage between 2 and 3, indicating moderate PD without severe motor complications;
  - Participants must exhibit mild cognitive impairment (MCI) according to criteria of Litvan et al. 2012 [36], ensuring that cognitive decline is present but does not meet the criteria for dementia;
  - No family history and no major cognitive impairment or major dysautonomic symptoms in history.
3. Medical stability
  - Participants must be on a stable dose of dopaminergic medications for at least three months prior to enrollment to reduce variability due to medication fluctuations;
  - If applicable, antidepressant or anxiolytic medications must be maintained at a stable dose for at least two months prior to study participation.
4. Comorbid conditions
  - Apart from clinical diagnosis of PD, participants must be in good health, with no history of uncontrolled chronic conditions that could interfere with the study outcomes;
  - Controlled comorbid conditions such as hypertensions or type 2 diabetes mellitus are permissible if they are well managed with medication and lifestyle measures.
5. Physical and functional status
  - Absence of significant postural instability or freezing-of-gait episodes that could interfere with participation in rTMS sessions.

#### 4.3.2. Exclusion Criteria

Participants will be excluded if they meet any of the following conditions:

1. Advanced disease stages
  - Hoehn and Yahr stages ≥ 4, indicating severe disease progression with significant motor and cognitive impairment.
2. Neurological and psychiatric comorbidities
  - Any neurological condition other than PD;
  - A history of major psychiatric disorders, including major depressive disorder (Beck Depression Inventory-II ≥ 29 [37] or active psychosis, which could confound cognitive assessments;
  - Transient ischemic attack, stroke, or any unexplained loss of consciousness or severe ongoing stressor within 1 year prior to screening.
3. Contraindications to rTMS
  - Contraindication to treatment will be investigated using a standard safety questionnaire to screen potential subjects for risk of adverse events during TMS (Safety Questionnaire for TMS [38]);
  - Presence of metallic implants, pacemaker, or other electronic medical devices that pose a safety risk during TMS session;
  - History of epilepsy or seizure disorders, as determined by self-reporting and medical history.
4. Others
  - Any medical conditions that are not stable or controlled, or, which in the opinion of the Investigator, could affect the subject’s safety or interfere with the study assessments and treatment;
  - Any medications that, in the opinion of the Investigator, may contribute to cognitive impairment, put the subject at higher risk for adverse events, or impair the subject’s ability to perform cognitive testing or complete study procedures.
  - Use of illicit narcotic medication.

Patients who fulfill the inclusion criteria and do not meet any exclusion criteria will be asked to enroll in the project.

Once enrolled, patients will be randomly assigned to one of three groups: (1) R-Gr, (2) S-Gr, or (3) C-Gr. A computerized random number generator (www.random.org/sequences/, accessed on 24 January 2025) with concealment, with a 1:1:1 allocation, will be used for randomization. Blood collection and neurocognitive assessment will be performed at baseline, T1, and T2.

The assessment will be conducted in one session and it will take 60 min for each patient. Schedule of study procedures is reported in Table 1.

### 4.4. Power Analysis

The primary outcome measures will be plasma BDNF, plasmatic EV-derived BDNF, and BADS. We anticipate positive score changes in the R-Gr group, a minimal effect in the S-Gr group, and, aside from a slight learning effect regarding cognitive performance, no changes in the C-Gr group. This will effectively balance out in our analyses because we will compare changes in means across these groups.

We have estimated our sample size using G*Power 3.1 for a repeated-measures MANOVA with between-group factors. The parameters were set as follows: medium effect size (f = 0.25), significance level (α = 0.05), power (1 − β = 0.80), correlation among repeated measure (r = 0.1), 3 groups, and 3 repeated measurements. The calculation indicated a required total sample size of 66 participants (approximately 22 per group) to detect a significant difference between groups. To account for a 10% dropout rate, the adjusted total sample size will be 73 participants (approximately 24–25 per group). This sample size will ensure adequate power to detect clinically meaningful differences while accounting for potential attrition.

### 4.5. Data Management

The data will be stored in REDCap (Harvard Catalyst, The Clinical and Translational Science Center). The project leader (GCR) will review it for correctness and plausibility in ca. 20% of the patients. The statistician will check the data also for plausibility before analyses.

## 5. Statistical Analyses

### 5.1. Primary Analyses

The baseline demographic, clinical, laboratory measures and cognitive scores will be compared between groups using an ANOVA for continuous variables and Chi-square tests for categorical variables. Covariates such as age and gender will be included if group differences exist at baseline.

A mixed-factorial ANOVA will be conducted to evaluate the effect of the rTMS protocol after 4 weeks of daily stimulation on plasma BDNF levels, the plasmatic EV-derived BDNF concentration, and EF performance. The model will include Group as a between-subjects factor and Time as a within-subjects factor.

### 5.2. Secondary Analyses

To assess the maintenance effect of rTMS 8 weeks post treatment on plasma BDNF, plasmatic EV-derived BDNF concentrations, and EF performance, separate mixed-factorial ANOVA models will be used. Group will serve as the between-subjects factor and Time (baseline, T2) as the within-subjects factor.

To evaluate the long-term effects of rTMS, a mixed-factorial ANOVA including Group as a between-subjects factor and Time (baseline, T1, T2) as a within-subjects factor will be performed. 

Relationships between plasma BDNF/plasmatic EV-derived BDNF values and EF performance scores post treatment (4 weeks) and at the 8-week follow-up will be explored using multivariate regression analysis, with adjustments made for confounders such as age, sex, and disease duration.

A Bonferroni correction will be applied to all mixed-factorial models to adjust for pairwise comparisons, minimizing the risk of the Type I error.

### 5.3. Interim Analysis

Data accumulation and treatment efficacy will be monitored through an interim analysis upon 50% enrollment to identify potential issues early.

## 6. Expected Results and Discussion

Increased plasma BDNF provides systematic neuroprotection, synaptic enhancement, and regeneration while plasma-derived exosomal EV BDNF delivers these effects with precision and stability to target regions. Together, they create a comprehensive and synergistic therapeutic framework for addressing a wide range of neuropathological conditions from neurodegenerative disease to acute brain injuries. PD is associated with decreased plasma BDNF levels, suggesting a potential link to the degeneration of dopaminergic neurons, while the findings regarding the exosomal BDNF levels present are inconsistent, showing, in several cases, that lower-plasma exosomal BDNF is associated with more severe motor symptoms in PD patients.

Thus, their deficiency may contribute to PD progression. Monitoring plasma and exosomal BDNF levels could aid in understanding disease mechanisms and evaluating therapeutic interventions aimed at increasing BDNF levels. A few protocols addressing intervention considerations to tempt reestablishing normal levels of plasma and exosomal BDNF levels in PD patients exist. However, we have evidence that has shown the impact of aerobic and resistance training to increase both plasma and exosomal BDNF levels, correlating with improvements in executive function tasks by enhancing prefrontal cortex plasticity [12].

Our protocol presents a novel and comprehensive approach to investigating the effects of rTMS on BDNF levels and EFs in PD patients. Three main points are highlighted to discuss the methodological rigor and expected outcomes of this study.

### 6.1. Innovative Design with Dual Control Groups

The inclusion of both sham and placebo control groups in the study design strengthens the ability to discern the specific effects of rTMS from placebo-induced dopamine release, a phenomenon often observed in PD patients due to heightened expectations of treatment efficacy [21,36]. Moreover, by employing a sham coil that replicates the noise and tactile sensations of real rTMS, the study minimizes the risk of participant unblinding, enhancing the internal validity of the findings [21]. Furthermore, the use of neuronavigation, guided by patient-specific MRI sequences, ensures the precise targeting of the DLPFC. This region is integral to EFs and dopaminergic activity, and its precise stimulation reduces variability and enhances the reproducibility of results [19,21]. 

### 6.2. Dual Measurement of Plasma BDNF and Plasmatic EV-Derived BDNF

The decision to measure both plasma BDNF and exosomal plasma-derived BDNF introduces a multidimensional approach to understanding the neurobiological effects of rTMS. Plasma BDNF levels provide systemic biomarkers of neurotrophic activity while exosomal BDNF reflects central-nervous-system-specific changes as exosomes can cross the blood–brain barrier and represent a neuronal origin [14,31]. This dual measurement strategy increases the validity and granularity of the findings, allowing for a more precise association between rTMS-induced changes in neurotrophins and cognitive outcomes.

Such an approach is supported by evidence demonstrating that rTMS increases BDNF levels both peripherally and centrally, contributing to synaptic plasticity and neuroprotection [12,15]. The integration of exosomal biomarkers in this study will enhance its translational potential, paving the way for biomarker-driven therapeutic strategies in neurodegenerative diseases.

### 6.3. Impact of rTMS on EFs Through Neurobiological Mechanism Modulation

EF is a hallmark of PD cognitive impairment that is closely linked to dopaminergic deficits in the fronto-striatal network [21]. High-frequency rTMS (HF-TMS) exerts beneficial effects on EFs in PD patients through multiple neurobiological mechanisms.

One of the primary mechanisms through which rTMS influences EF is by enhancing neuroplasticity and synaptic modulation. BDNF is a key regulator of synaptic strength and plasticity, particularly within the prefrontal–striatal circuitry, which is critically affected in PD. Increased BDNF expression induced by rTMS may facilitate long-term potentiation (LTP) in the DLPFC, thereby improving cognitive processes such as working memory, cognitive flexibility, and attention. 

Additionally, rTMS interacts with fronto-striatal and dopaminergic pathways, which are central to cognitive and motor control in PD. Studies have demonstrated that HF-rTMS’s stimulation of the DLPFC enhances dopamine release within the striatum [39], indirectly promoting BDNF expression and improving executive function [40]. This dopaminergic enhancement may help restore the functional integrity of neural circuits underlying cognitive performance in PD.

A novel aspect of our protocol is the investigation of exosomal BDNF as a neuroprotective biomarker of rTMS effects. Exosomes, which are capable of crossing the blood–brain barrier, serve as critical carriers of neurotrophic factors such as BDNF. rTMS may promote the secretion of BDNF-containing exosomes, providing targeted neurotrophic support to brain regions affected by PD. The observed increase in plasma-derived exosomal BDNF following rTMS suggests a potential mechanism by which neuroplastic changes in the brain may be reflected in peripheral biomarkers [41].

Furthermore, rTMS has been shown to mitigate neuroinflammation [42] and oxidative stress [43,44], both of which contribute to PD progression and cognitive decline [44]. By reducing pro-inflammatory cytokine levels and oxidative damage, rTMS may create a neuroprotective environment that enhances the effectiveness of BDNF in supporting neuronal health and cognitive function.

Lastly, the circuit-specific effects of rTMS on the bilateral DLPFC play a crucial role in improving EF. The DLPFC is a key hub within the frontoparietal network, which governs cognitive control and decision-making. rTMS-induced plasticity within this region may enhance connectivity and functional outcomes, thereby alleviating EF deficits commonly observed in PD.

These findings collectively suggest that rTMS provides a non-invasive therapeutic approach to modulate BDNF-related mechanisms, thereby improving both motor and cognitive symptoms in PD. 

An aspect not considered in the protocol is the examination of BDNF variations at different stages of PD, although previous findings have shown that BDNF levels decline as the disease progresses and shown its correlation with clinical severity in neurodegenerative diseases [45,46]. However, limiting the enrollment of subjects with mild–moderate severity of disease was decided upon to avoid confounders that could alter the recording of the treatment’s effectiveness. Future research should investigate rTMS-induced BDNF changes across different disease stages and their correlation with clinical outcomes.

The proposed protocol, while theoretical, is grounded in robust evidence from preclinical and clinical studies that have demonstrated the neurotrophic and cognitive benefits of rTMS. Currently, we have initiated patient recruitment and finalized the logistical framework for conducting the study, including neurocognitive assessments, blood sampling, and biomarker analysis. 

Although preliminary findings are unavailable at this stage, the theoretical framework is strengthened by prior studies indicating that high-frequency rTMS targeting the DLPFC increases plasma and exosomal BDNF levels, which correlate with improved EFs in neurodegenerative conditions. 

This protocol represents an essential step toward validating rTMS as a non-invasive intervention for Parkinson’s-disease-related cognitive impairments.

## 7. Conclusions

rTMS promotes the reestablishment of plasma and exosomal BDNF levels through mechanisms such as synaptic plasticity, neurogenesis, and anti-inflammatory modulation. These changes enhance executive functioning by optimizing prefrontal–striatal circuitry and supporting dopaminergic systems. The clinical and research implications of these findings position rTMS as a promising therapeutic modality for addressing both cognitive and motor challenges in PD. 

The excepted findings may lie in enhancing our understanding of neuroplasticity while their clinical relevance includes their providing a non-invasive, neuroprotective, and individualized treatment option. Integrating rTMS into research and clinical practice has the potential to transform care for PD and other neurological disorders.

### Impact Statement

This protocol introduces a pioneering approach to addressing cognitive impairments in PD by leveraging rTMS. By exploring plasma and exosomal BDNF as dual biomarkers, the study will bridge the gap between non-invasive neurostimulation techniques and targeted therapeutic strategies for EF.

The findings could catalyze broader applications of rTMS in treating neurodegenerative diseases, advancing the field of translational medicine and improving the quality of life for patients.

## Figures and Tables

**Table 1:** Overview of schedule protocol’s procedures established for each time point of protocol.

Time (week)	−2	0		+4	+12
Visit	Screening	T0	rTMS (4 Weeks)	T1	T2
Inclusion/exclusion criteria	*				
Informed consent signature	*				
Demographic and clinical data	*				
MoCA	*				
Beck Depression Inventory	*				
SQ TMS	*				
**Laboratory analysis** Blood collection		*		*	*
**Neurocognitive** **Assessment** BADS PDSQ-39		*		*	*
**Neurostimulation Treatment** rTMS			▲		

rTMS: repetitive transcranial magnetic stimulation; T0: baseline visit; T1: visit after 4 weeks of rTMS treatment; T2: visit after 8 weeks, at the end of rTMS treatment; SQ TMS: Safety Questionnaire for TMS; MoCA: Montreal Cognitive Assessment; BADS: Behavioural Assessment of the Dysexecutive Syndrome; PDQ-39: Parkinson’s Disease Questionnaire; ▲: rTMS intervention.

## Data Availability

The original contributions presented in this study are included in the article. Further inquiries can be directed to the corresponding author.

## Abbreviations

AD	Alzheimer’s disease
ANOVA	Analysis of Variance
APC	Allophycocyanin
BADS	Behavioural Assessment of the Dysexecutive Syndrome
BDNF	Brain-Derived Neurotrophic Factor
CD	Cluster of Differentiation
C-Gr	Control Group
DA	Dopamine
DLPFC	Dorsolateral Prefrontal Cortex
DLS	Dynamic Light Scattering
EF	Executive Function
EV	Extracellular Vesicle
HF	High Frequency
MANOVA	Multivariate Analysis of Variance
MCI	Mild Cognitive Impairment
MR	Magnetic Resonance
MRI	Magnetic Resonance Imaging
mRNA	messenger Ribonucleic Acid
MT	Motor Threshold
nMFI	Normalized Median Fluorescence Intensity
NTA	Nanoparticle Tracking Analysis
PD	Parkinson Disease
PDQ-39	Parkinson’s Disease Questionnaire
REDCap	Research Electronic Data Capture
R-Gr	Real Group
rTMS	repetitive Transcranial Magnetic Stimulation
S-Gr	Sham Group
TMS	Transcranial Magnetic Stimulation
